# Non-Genomic Hallmarks of Aging—The Review

**DOI:** 10.3390/ijms242015468

**Published:** 2023-10-23

**Authors:** Drahomira Holmannova, Pavel Borsky, Helena Parova, Tereza Stverakova, Milan Vosmik, Libor Hruska, Zdenek Fiala, Lenka Borska

**Affiliations:** 1 Institute of Preventive Medicine, Faculty of Medicine in Hradec Kralove, Charles University, 500 03 Hradec Kralove, Czech Republic; holmd9ar@lfhk.cuni.cz (D.H.); fiala@lfhk.cuni.cz (Z.F.); borka@lfhk.cuni.cz (L.B.); 2 Department of Clinical Biochemistry and Diagnostics, University Hospital, Faculty of Medicine in Hradec Kralove, Charles University, 500 03 Hradec Kralove, Czech Republic; helena.parova@fnhk.cz (H.P.); stverat@lfhk.cuni.cz (T.S.); 3 Department of Oncology and Radiotherapy, University Hospital, Faculty of Medicine in Hradec Kralove, Charles University, 500 03 Hradec Kralove, Czech Republic; vosmik@fnhk.cz (M.V.); libor.hruska@fnhk.cz (L.H.)

**Keywords:** aging, hallmarks, non-genomic, cancer

## Abstract

Aging is a natural, gradual, and inevitable process associated with a series of changes at the molecular, cellular, and tissue levels that can lead to an increased risk of many diseases, including cancer. The most significant changes at the genomic level (DNA damage, telomere shortening, epigenetic changes) and non-genomic changes are referred to as hallmarks of aging. The hallmarks of aging and cancer are intertwined. Many studies have focused on genomic hallmarks, but non-genomic hallmarks are also important and may additionally cause genomic damage and increase the expression of genomic hallmarks. Understanding the non-genomic hallmarks of aging and cancer, and how they are intertwined, may lead to the development of approaches that could influence these hallmarks and thus function not only to slow aging but also to prevent cancer. In this review, we focus on non-genomic changes. We discuss cell senescence, disruption of proteostasis, deregualation of nutrient sensing, dysregulation of immune system function, intercellular communication, mitochondrial dysfunction, stem cell exhaustion and dysbiosis.

## 1. Introduction

The global population is gradually aging. This is often unhealthy aging, which is associated with significant polymorbidity and premature deaths. However, it also has other negative effects, such as the reduced participation of older people in the labor market, increased medical costs, and full dependence on the help of others. *The World Social Report 2023: Leaving No One Behind in an Ageing World* by the United Nations describes the current and future state of the aging population as follows: the number of people aged 65 and older worldwide will more than double, from 761 million in 2021 to 1.6 billion in 2050 [1,2].

As the population ages, the prevalence of cancer also increases [3]. However, cancers are also becoming more common in younger people. Despite advances in diagnosis and treatment, cancer is the second most common cause of premature death worldwide. Many factors play a role in the pathogenesis of cancer. Although much attention has been paid to changes in the genome, non-genomic changes are also important. Although there are no overlaps between aging and cancer in terms of genomic changes, there are in non-genomic changes. Controlling non-genomic hallmarks of aging could also lead to cancer prevention.

Aging is defined as a process in which there is a gradual loss of organ function and a reduction in the ability to regenerate. This is due to the multiple changes that occur at the molecular and cellular levels. Various theories have been formulated to explain the cause of aging, e.g., oxidative damage or programmed theory. These theories cover only a certain aspect of the aging process and do not consider its full complexity. The theory that connects all causes of aging is based on the hallmarks of aging, molecular processes that accumulate damage during aging that exceed the cell’s ability to repair it [4].

**Figure 1:**
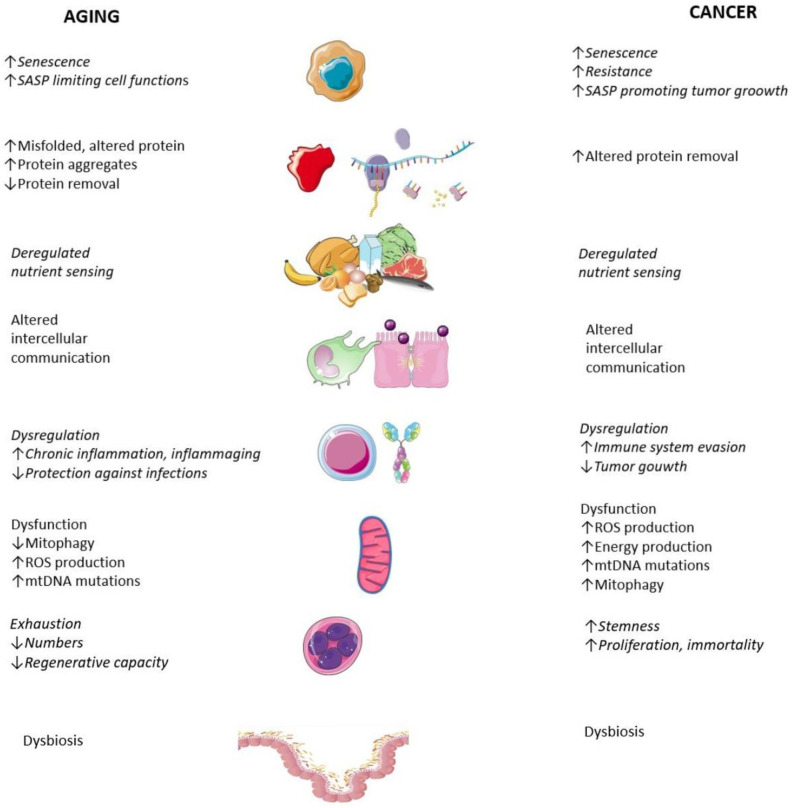
Hallmarks of aging and cancer.

Hallmarks of aging include the following factors, which can be detected using specific markers. The hallmarks of aging can be divided into genomic hallmarks (closely related to DNA) and non-genomic hallmarks. Genomic hallmarks include genomic instability, telomere attrition and epigenetic alterations. Non-genomic hallmarks include cellular senescence, loss of proteostasis, deregulated nutrient sensing, altered intercellular communication, immune system dysfunction and chronic inflammation, stem cell exhaustion and mitochondrial dysfunction ad dysbiosis (Figure 1).

Cancer is a process by which a cell originally inherent in the body gains an evolutionary advantage and begins to outgrow its surroundings and later its host. The basis for gaining this advantage is the presence of genetic or epigenetic changes. Neoplastic cells are subsequently divided into cell clones, including further dedifferentiation, increasing malignant potential and gaining the typical hallmarks of cancer described earlier by Hannahan and Weinberg (Figure 1) [5,6,7]. The traditional concept of carcinogenesis speaks of the presence of at least two crucial DNA mutations. Genes whose genetic or epigenetic changes lead to malignant cell transformation are divided into oncogenes and tumor suppressor genes. Only 5–10% of tumors are caused by inherited germline mutations; the rest are due to somatic mutations occurring during life. Carcinogenesis consists of three phases: initiation, promotion and progression [8].

Different processes and mechanisms based on theories describing aging operate at different levels of carcinogenesis. The correlation between exposure to a particular carcinogen and the duration of exposure increases with age; therefore, the increased likelihood of a mutation determining tumor development is now an accepted fact. Traditional cytostatic treatment is based on damaging the genetic information of the cancer cell, which leads to cell death. In contrast, modern, targeted anticancer therapies focus on affecting the tumor microenvironment (angiogenesis, immune system, etc.), cellular signaling or tumor organelles and cellular components other than tumor DNA. Understanding the link between non-genomic influences on the cell in the aging process and carcinogenesis could have outcomes for both understanding the actual malignant transformation and the impact on targeted anticancer therapy [6].

For our review, we have decided to cover the current state of knowledge in all non-genomic hallmarks of aging. To further illustrate, we explore the basic principles in which cancer, as an example of age-related diseases, intertwines with the hallmarks of aging. The review is divided into eight main chapters: senescence, disrupted proteostasis, deregulated nutrient sensing, altered intercellular communications, immune system dysfunction, mitochondrial dysfunctions and dysbiosis.

The hallmarks of aging and cancer largely coincide. The difference is due to the changes that occur in each hallmark.

## 2. Senescence

Cellular senescence is considered one of the main signs of aging. It is characterized by irreversible cell cycle arrest (despite optimal conditions) and impaired function and intercellular communication, which can promote inflammation and compromise other cells and tissues [9]. Senescence occurs in response to various stress stimuli, such as DNA damage, telomere shortening, oncogene activation, oxidative stress and paracrine secretion (reinforcement of senescence). These senescence triggers are associated with aging, inflammation or carcinogenesis and at the same time further promote these pathological processes. Senescence affects all cell types, including immune and stem cells [10].

Crucial non-genomic markers of senescence include [11] the senescence-associated secretory phenotype (SASP), which is constituted of the expression of the tumor suppressors and cell cycle regulators p16ink4a, p21 and p53, the accumulation of β-galactosidase in lysosomes and the accumulation of lipofuscin.

### 2.1. Senescence and the SASP Phenotype in Aging and Cancer

One of the typical and important features of senescent cells is a change in the phenotype into SASP. The SASP transcriptional program is responsible for the production of multiple cytokines, chemokines, growth factors, extracellular matrix proteases, etc., and is involved in aging and cancer progression. SASP can transfer senescence from cell to cell.

The composition of SASP varies depending on the cell type and the nature of the initial stimulus. However, there is a core program that is identical between cells and independent of the stimulus that induces senescence comprising mainly proinflammatory substances, such as IL-6, IL-8 and MCP1 (monocyte chemoattractant protein 1) [12].

Senescent cells have a multitude of functions; some can be detrimental, others beneficial. Beneficial functions include the promotion of embryogenesis and placentogenesis, the limitation of excessive cell proliferation during regeneration, the promotion of wound healing and tumor suppression. The induction of senescence by SASP in precancerous cells and their elimination by immune cells is a prevention of the early onset of cancer. However, senescent cells can also inflict a range of damage. They promote a proinflammatory microenvironment and immune system dysregulation. Furthermore, SASP supports tumor development and progression. The proinflammatory microenvironment and impaired antitumor immunity facilitate tumor cell proliferation and growth. SASP matrix metalloproteinases destruct the tissue architecture and allow tumor cells to migrate and invade the surrounding tissue [10,13]. SASP induces senescence in stem cells, leading to a complete loss of their regenerative potential. Their loss of regenerative potential will be discussed in the dedicated section [14]. On the other hand, the study by Milanovic et al. shows that the SASP phenotype can induce stemness in cancer cells and thus promotes cancer progression [15]. Even senescent cancer stem cells play a role in tumor progression and, more importantly, the resistance to treatment [16].

### 2.2. Senescence and the Expression of p16 and p21 in Aging and Cancer

Another very crucial sign of senescence, besides the SASP phenotype, is the expression of tumor suppressors and cell cycle regulators e.g., p16INK4A (p16) and p21. Multiple studies on aging and cancer have confirmed changes in the expression of these markers. Although an increase in the expression of these markers is typical for aging, the situation in cancer varies according to the type of tumor.

In studies comparing the expression of individual parameters between subjects of different age groups (younger vs. older), increased expression of p16 and p21 has been demonstrated in many tissue types. This is associated with a decline in organ functions. Changes in p16 and p21 expression in the skin are well described. The expression of p16 increases and the number of p16-positive cells is correlated with age in both the dermis and epidermis and can serve as a robust marker of cellular aging in the skin [17,18]. The increase in the expression of p21 in older people interferes with the regenerative process during wound healing [19]. Changes in p16 and p21 expression due to aging have also been detected in other tissues.

Idda et al. mapped the expression of senescence markers in distinct human tissues. The number of cells expressing p16 and p21 increased with age in the epidermis, pancreas and kidney. Cells expressing p16 were abundant in the brain cortex, liver, spleen and colon and p21 cells increased in the dermis, whereas the number of cells expressing both proteins in the lung did not change with age. Surprisingly, the authors did not detect cells positive for p21 and p16 in muscle [20].

Although Idda et al. did not detect changes in p16 expression in lung and skeletal muscle, Wicher et al. demonstrated that declination in lung function during aging is associated with the presence of senescence in airway smooth muscle and extracellular matrix deposition. They examined samples from individuals and measured markers of senescence and changes in intracellular calcium signaling and extracellular matrix. Production of p21, γH2AX and β-gal and SASP markers increased with aging [21].

Senescence plays a dual role in the development and progression of tumors. It can act as a preventive measure for tumor formation but also as a driver of pathological processes. Cancers are a highly heterogeneous group, not only because they affect different tissues but also because heterogeneity is evident between tumors of the same tissue. In certain tumors and at different stages and grades of the same tumor, we encounter both an increase and a decrease in senescence markers [22].

Since senescence markers block cell division, it is more common that their expression is reduced in tumor cells and cancer cells that can proliferate. Several anticancer therapies are based on the induction of senescence in cancer cells. On the other hand, senescence can promote tumor growth. SASP factors induce inflammation, mitogenic signaling, stemness, angiogenesis, genotoxicity and finally immunosuppression. Anticancer treatment induces senescence not only in cancer cells but also in noncancer cells; senolytics may help with the treatment of cancer [23]. As the situation in cancer is more intricate than in aging, we will mention some studies in more detail.

First, we focus on solid tumors. Increased expression of p16 can be a risk factor for cancer, such as skin tumors, breast cancer or cervical cancer; furthermore, the expression can reflect the aggressiveness of tumor [24,25,26]. However, several studies have described a decrease in the levels of p16 in tumor cells, e.g., in laryngeal squamous cell carcinoma [26]. p16 is evaluated not only in solid but also in hematologic malignancies such as acute myeloid leukemia (AML) and acute lymphoid leukemia (ALL).

Taniguchi et al. described the expression of p16 and p14ARF in various types of hematologic malignancies including AML, ALL, follicular lymphoma and diffuse large B-cell lymphoma. Patients with ALL, AML or blastic crisis chronic myelogenous leukemia expressed higher levels of p16 mRNA more often than patients with other diseases [27]. In the mouse AML model, the reduction of p16 expression can improve survival [28].

The p21 marker is also of interest to scientists in the context of senescence and cancer. Huang et al. investigated the role of the subcellular expression of p21 in gastric cancer. Nuclear p21 levels were reduced, whereas cytoplasmic levels increased compared with non-cancerous tissues. The authors described that nuclear p21 inhibits but cytoplasmic p21 drives cell migration and invasivity. High cytoplasmic levels of p21 correlated with the severity of cancer and shorter overall survival [29].

Increased senescence is associated with both aging and carcinogenesis. Targeting this phenomenon, the use of senolytics may be beneficial in slowing the aging process as well as reducing the risk of cancer.

## 3. Proteostasis

Proteostasis is essential for the proper functioning of any cell. It is a complex of processes that maintain proteome homeostasis, including protein synthesis, folding, assembly, turnover and degradation. Proteostasis is disturbed during aging. Any step involved in proteostasis can be disrupted. This leads to the production of dysfunctional proteins and insufficient elimination and accumulation of proteins in the cell and its organelles, e.g., the endoplasmic reticulum [30].

### 3.1. Protein Synthesis and Quality Control

Protein synthesis depends on the quality of the mRNA transcript and ribosomal functions (translation). Aging is associated with genomic changes, DNA damage and epigenetic modifications that can lead to the alteration of transcription. Transcription of certain genes can be silenced, whereas others can be transcribed at a more extensive level [31]. There is also a decrease in ribosome function, translation (initiation and elongation) and the quality control of synthesized proteins [30,32].

**Figure 2:**
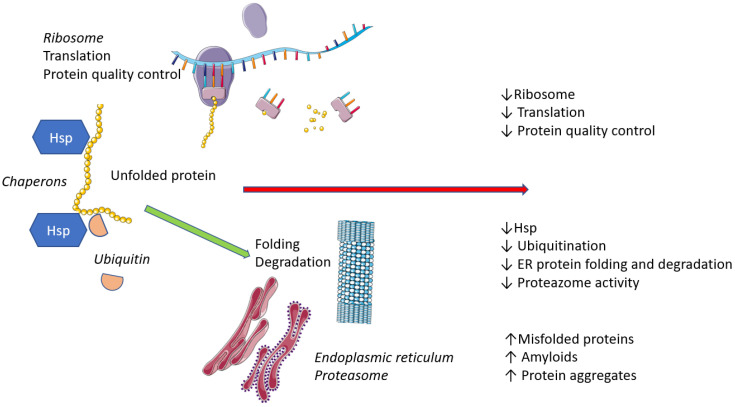
Protein control mechanisms. Legend: The ribosome translates the template mRNA into a polypeptide chain (unfolded protein) that is subsequently bound by heat shock proteins (Hsp) and ubiquitinases, allowing the chain to be modified and transported to the endoplasmic reticulum (ER). Here, they are processed into final functional proteins or degraded. Aging is associated with a reduction in ribosome number, translation and the quality control of produced proteins. Transfer of proteins to the ER and ER functions are also limited, which leads to accumulation of defective, unfunctional proteins.

The synthesized protein chain undergoes conformational changes, i.e., folding, in which chaperons and the endoplasmic reticulum are involved. During folding, the protein gains stability and its specific function. Aging is associated with increased endoplasmic reticular stress and increased production of unfolded or misfolded proteins (Figure 2) [33].

### 3.2. Protein Degradation

We have briefly mentioned the first step and will focus more on the degradation of dysfunctional proteins. Several mechanisms are involved in protein degradation. The most important are autophagy (autophagic lysosomal degradation) and the ubiquitin–proteasome system (UPS) and the processes occurring in the endoplasmic reticulum, the unfolded protein response (UPR) and endoplasmic-reticulum-associated degradation (ERAD). All these protein degradation processes are altered during aging and tumorigenesis. Aging is associated with a decrease in the efficiency of protein degradation and protein-accumulation-induced proteotoxicity [34,35,36,37]. On the other hand, in cancer, the activity and efficiency of protein degradation processes are increased and serve as protection against cell damage [38,39]. We will describe autophagy and its role in aging and cancer in more detail.

Autophagy is an intracellular multistep process responsible for the catabolic degradation of cytoplasmic macromolecules, protein aggregates, damaged organelles (mitophagy, lysophagy), pathogens and even whole cells (cell death type). It is essential to maintain protein homeostasis, reduce oxidative stress, maintain immune memory, nutrient-sensing, cell survival, preserve genome integrity, etc. It can be induced by various stimuli, such as oxidative stress, inflammation, cellular starvation/nutrient depletion, the presence of pathological cytoplasmic material and mitochondrial of endoplasmic reticulum stress [40,41].

Autophagy is known to decrease during aging and maintenance of autophagy, or its induction, can delay aging and prolong lifespan, as we see in laboratory animals [42]. The importance of maintaining or inducing autophagy is demonstrated through various studies. Activation of autophagy in adult animals can prevent an age-related decline in brain functions and stimulate neurogenesis and axonal regeneration after injury [43,44]. It can also promote self-renewal of the salivary gland and liver or muscle regeneration, so autophagy activators could be promising anti-aging therapeutics and could even find potential in the treatment of myopathies [42,45,46]. Interestingly, autophagy can be induced by a well-known drug used in the treatment of type II diabetes, metformin [47].

Human studies determining the assembly of autophagosomes and their fusion with lysosomes analyze autophagy markers that reflect a particular phase of the autophagy process (LC3, p62, Atg members, LAMP2). The levels of markers reflecting autophagosome formation are decreased in older people compared with younger people [48]. The levels of a marker responsible for autophagosome–lysosome fusion (LAMP2) are also reduced. Thus, paradoxically, autophagosomes may accumulate in cells since they are not degraded after fusion with the lysosome [49].

**Figure 3:**
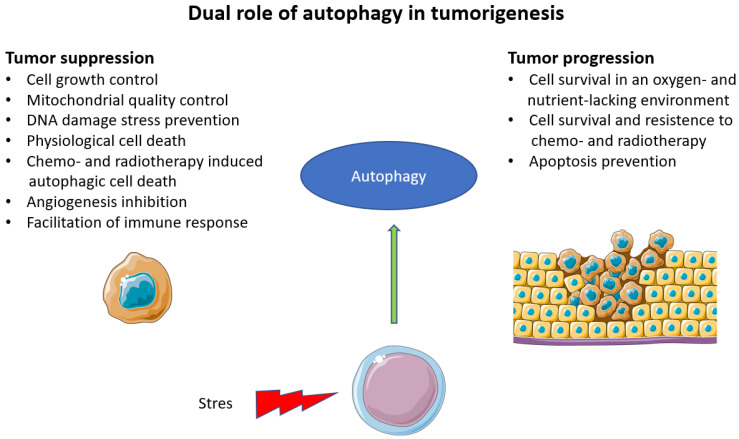
Dual role of autophagy in tumorigenesis. Legend: autophagy has a dual role in tumorigenesis. It controls cell growth, mitochondrial health, prevents DNA damage, mediates physiological cell death, induces cell death induced by external factors such as anticancer therapy, inhibits neovascularization and enhances immune system function and antitumor immunity; in contrast, autophagy is also responsible for the increased survival of cancer cells in environments with low levels of oxygen and nutrients, induces resistance to anticancer therapy and protects cells from apoptosis.

Autophagy is also closely related to cancer (Figure 3). In a similar way to aging, the activation of autophagy is often diminished. The basal level of autophagy functions as a mechanism of tumor suppression by reducing damaged cellular parts and proteins and maintaining cellular homeostasis. It also reduces invasivity and metastasis and maintains sensitivity to treatment. Disruption of autophagy, therefore, allows accumulation of pathologies in the cell and facilitates malignant transformation, cell proliferation and survival. Autophagy can be a therapeutic target [50]. However, autophagy can also drive tumorigenesis.

The tumor microenvironment is characterized by a lack of nutrients and oxygen. This triggers autophagy, which helps meet nutritional demands and maintain viability and proliferation. Thus, changes in autophagy occur in cancer cells depending on the environment in which the cell is located. Increased autophagy also ensures the removal of potentially pathogenic proteins and prevents their accumulation. Although a decrease in autophagy in the early stages can promote tumor progression, an increase in the later stages can lead to survival and resistance of the cancer cell to treatment [51,52]. Autophagy markers such as LC3, members of the Atg family or p62 can serve as prognostic factors in various types of tumors, including both solid and hematologic malignancies. Elevation of p62 in patients with non-small cell lung cancer, glioma or gastric cancer is associated with proliferation, migration, tumor invasivity, resistance to chemotherapy and poor prognosis [53,54,55].

The results obtained from studies on colorectal cancer have shown the opposite. Higher levels of LC3 and p62 are associated with better overall survival.

Mohamadimaram et al. tested the expression of the Atg7 and LC3 genes in patients with acute myeloid leukemia. The decrease in LC3 expression was 75.55%. Interestingly, LC3 overexpression was detected in 11.33% of 55 patients [56].

As is evident from these few studies cited, changes in autophagy, and thus changes in the expression of mRNAs and proteins related to autophagy, occur in cancer. These changes, which can be easily detected, are specific to certain types and can be used as markers to monitor cancer progression and the presence of resistance to treatment.

### 3.3. Posttranslational Protein Modifications—AGEs

When describing proteostasis, we cannot avoid the topic of the pathological modification of proteins, which changes the nature and function of proteins and can activate inflammatory reactions and the production of reactive oxygen species. The most significant process is glycation.

Glycation is a non-enzymatic process in which glucose, or another carbohydrate, binds to a protein, nucleoside or lipid. Glycation-modified compounds are referred to as AGEs (advanced glycation end products). DNA is also very sensitive to glycation, especially in the presence of glyoxal and methylglyoxal, resulting in so-called nucleotide AGEs [57].

AGEs in the organism have a dual origin. They can be exogenous from the food we consume or endogenous, those that are formed in the body, for example, in conditions with higher glucose concentrations and during nonpathological metabolic processes [57,58,59].

AGEs can accumulate both intracellularly and extracellularly. Intracellular AGEs negatively affect cell functions, can stimulate aberrant protein glycation, cause abnormal protein folding, protein aggregation and increase oxidative stress and activate signaling pathways associated with inflammation and apoptosis, mitochondrial and endoplasmic reticular stress. Cross-linking of AGE proteins in the mitochondrial respiratory chain disrupts the production of ATP [60].

The concern is not only intracellular AGEs but also extracellular ones. Glycated proteins are also proteins with long half-lives, such as laminin, collagen or elastin, and form part of many tissues, including blood vessels, muscle, cartilage, eye lens, etc. We cannot ignore the fact that IgG antibodies are also glycated, thus changing their function and their ability to bind antigen and induce effector cell activity. AGEs bind to their RAGE receptors, triggering intracellular JNK and ERK/MAPKp38 signaling pathways with activation of NF-κB transcription factors, AP-1 and STAT3. This increases the production of pro-inflammatory cytokines [61,62,63].

The concentration of AGEs in the body depends not only on uptake and production but also on degradation. The glyoxalase system is responsible for this: glyoxalase 1, glyoxalase 2 and the cofactor glutathione [64].

Aging is associated with increased levels of AGEs that depend on the increased production and degradation activity of glyoxalases [64,65,66].

The involvement of AGEs and RAGEs in the aging process is indisputable. These molecules also play an important role in carcinogenesis. Their importance in carcinogenesis is reflected by the fact that many researchers have described that AGEs and RAGEs can serve as markers of the presence and progression of tumors and, at the same time, these molecules have become targets for anticancer therapy. Cancer cells show a higher expression of RAGEs and AGEs [67,68].

Peng et al. measured the levels of AGEs and RAGEs in patients with breast carcinoma and controls. Higher levels of AGEs and the AGEs/RAGE ratio were associated with an increased risk of breast cancer. The authors also documented a positive association between AGEs and poor prognosis [69].

Maintaining proteostasis, the balance between protein production and degradation, is essential to maintain health and reduce the risk of age-related disease, e.g., cancer. Its disruption accelerates aging, the development of chronic diseases and cancer.

## 4. Deregulated Nutrient Sensing

To describe the issue of deregulation of nutrient sensing, we must first explain what nutrient sensing is.

Nutrient sensing is the ability of a cell to sense and respond to fluctuations in the levels of nutrients, the substrates necessary for survival. Each nutrient (glucose, fatty acids, amino acids, etc.) is sensed by different sensors and processed through different pathways. Nutrient sensors and their signaling regulate the extension of lifespan, since various sensors are products of “longevity genes”. These pathways are deregulated in aging cells, in metabolic diseases and, evidently, in cancer [70].

There are four main nutrient-sensing pathways that are strongly influenced by aging. They are associated with insulin-like growth factor 1 (IGF-1), mammalian target of rapamycin (mTOR), sirtuins and AMP-activated kinase (AMPK). Several nutrients can activate different pathways.

### 4.1. Insulin/IGF-1 → PI3K/AKT and mTOR

**Figure 4:**
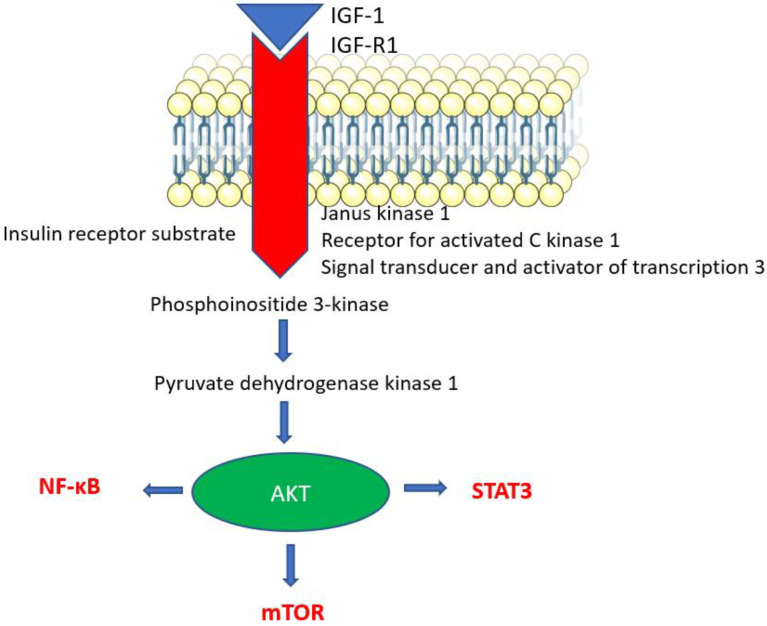
mTOR activation pathway. Legend: The IGF1 pathway: the receptor for IGF-1 (insulin growth factor 1), IGF-R1, is associated with molecules such as Janus kinase 1, insulin receptor substrate, etc.; its activation results in the activation of phosphoinositide 3-kinase, which activates pyruvate dehydrogenase kinase 1 and then AKT (protein kinase B) is activated. AKT can induce the activation of the nuclear transcription factor NF-κB and transcriptional factor STAT3 and therefore induce the production of inflammatory cytokines and mTOR (mammalian target of rapamycin).

Insulin and IGF-1 levels increase when glucose levels are elevated and then the expression of the IGF-1 receptor increases (Figure 4). The involvement of IGF-1 with its receptor activates PI3K, and PI3K in turn activates AKT, which phosphorylates and activates mTOR and inhibits FOXO. These signaling pathways play an important role in many processes (cell growth, survival, metabolism) by triggering various intracellular processes [70]. Disruption of the insulin/IGF-1 signaling pathway is associated with several diseases (cardiovascular and neurodegenerative diseases, type II, cancer) and aging. Various in vivo studies in *Caenorhabditis elagans*, *Drosophila melanogaster* or rodents showed that a loss of function mutation or blockade of members of the insulin/IGF-1 pathway can prolong life [71,72,73]. However, the results of these preclinical studies are not in full agreement with the results of clinical studies. In studies with centenarians, which are an appropriate model of aging, elevated and lower IGF-1 levels have been measured. High and low levels are associated with insulin resistance [74]. This reflects the complexity of this pathway and its involvement in many processes in the body [73].

In general, IGF-1 levels decrease with age [75]. Milman and Vitale et al. evaluated IGF-1 levels in centenarians, who have lower levels of IGF-1 than elderly non-centenarian people [76,77]. Paolisso et al. confirmed that IGF-1 levels declined dramatically until 74 years of age, after which the decline stopped (75 to 99 years). However, in centenarians, there is a decline in IGFBP-3 (insulin-like growth factor binding protein 3) and there is an increase in the IGF-1/IGFBP-3 ratio that improves insulin sensitivity [78]. Lower activity of the IGF-1 pathway is also detected in the children of centenarians compared with age-matched controls [79]. The results suggest that a decline in IGF-1 with age is desirable but a certain level must be maintained, which is what happens in centenarians. The levels do not drop further.

Importantly, the insulin/IGF-1 pathway is regulated by miRNA (nutrimiRNA and nutrimiRNA aging) and the let-7 miRNA family, which can regulate activity of any member of this pathway and improve insulin sensitivity. For example, miRNA-1 is upregulated in progeria livers in models and regulates IGF-1/PI3K/AKT/mTOR; miRNA-383 is also upregulated and targets IGF-1 and its receptor [80].

mTOR is considered a master regulator of aging and a negative regulator of lifespan. TOR is a serine/threonine kinase with multiple functions. It also senses nutrients and plays a role in senescence, proteostasis maintenance, mitochondrial functions, etc., [81].

A variety of in vivo studies have demonstrated the effect of mTOR in aging, e.g., Wang et al. evaluated the expression and activity of proteins involved in the activation of mTOR in a zebrafish model. Expression of mTOR and AKT was elevated in aging animals compared with young animals [82]. An anti-aging effect for rapamycin administration, which blocks mTOR, has also been described, even in progeroid mice, and this may alleviate age-related changes in progenitors [83,84].

Proteins belonging to the insulin/IGF-1 pathway are also deregulated in cancer. IGF-1 acts as a growth factor; thus, it has the capacity to induce proliferation, survival, and inhibit cell apoptosis. Furthermore, it suppresses antitumor immunity through the STAT3 pathway [85]. An association between IGF-1 levels and cancer risk has been demonstrated. Several studies are worth mentioning, e.g., the study by Murphy et al., who analyzed data from 397,380 participants and evaluated whether levels of IGF-1 and IGFBP3 were associated with the risk of colorectal cancer. They found that higher levels of IGF-1 and IGFB3 were associated with the risk of colorectal cancer [86].

### 4.2. AMPK ↔ SIRT

**Figure 5:**
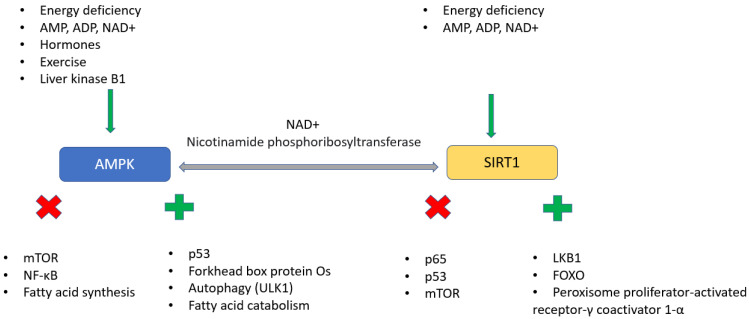
AMPK and SIRT1. Legend: AMPK (adenosine monophosphate activated protein kinase) and SIRT1 (sirtuin 1) activated by specific stimuli (green outer arrows); both can activate each other (grey double arrow). AMPK and SIRT1 support expression and activity of different proteins and genes (green plus), whereas they also inhibit other proteins and genes (red x). Abbr.: NAD, nicotinamide adenine dinucleotide; AMP, adenosine monophosphate; ADP, adenosine diphosphate; LKB1, serine-threonine kinase 11; FOXO, forkhead box O; ULK1, unc-51-like kinase 1; mTOR, mammalian target of rapamycin.

AMPK (adenosine monophosphate activated protein kinase) activity is regulated by hormonal and metabolic signals, for example, glycemia, and is a natural sensor of the amount of energy in the cell. It is activated when intracellular adenosine monophosphate and adenosine diphosphate levels increase following a decrease in ATP levels. Activated AMPK has a wide functional range, controls autophagy, participates in fatty acid catabolism and glycolysis, blocks mTOR, reduces glucose uptake or inhibits NF-κB. During aging, AMPK activity decreases, and preservation of its activity extends lifespan. Metformin has been found to activate the AMPK/brain-derived neurotrophic factor/PI3K pathway and alleviate the neurocognitive decline associated with aging. A similar anti-aging effect also has polyphenols that activate AMPK. It is important to mention that the decrease in AMPK can be alleviated by physical activity (Figure 5) [87,88,89,90].

In the context of AMPK, we also mention sirtuins, which are NAD^+^ deacetylases that mutually activate each other. In this regard, SIRT1 is the most involved, responding to the amount of nutrients but also to NAD^+^ levels, which, as we have already mentioned, decrease with age. Therefore, the concentration and activity of SIRT1 also decrease. Sirtuin activity delays telomere shortening, promotes the maintenance of genome integrity, enhances DNA repair, promotes autophagy, ameliorates insulin resistance, maintains an impermeable blood–brain barrier, can mitigate age-related senescence in stem cells, etc., [91,92,93,94].

Kilic et al. described *SIRT1* gene polymorphisms and their association with aging. The highest levels of SIRT1 are in the elderly with the genotype rs7895833, which was associated with a lower level of oxidative stress [95].

De Arellano et al. measured the levels of SIRTT1 and SIRT 3 in human ventricular tissue in young (17 to 40 years) and old (50 to 68 years) participants. In the hearts of older women, the expression of both sirtuins was significantly lower than in younger women. It was associated with a decrease in the expression of the antioxidative protein SOD2 and infiltration of heart tissue with macrophages and the production of pro-inflammatory cytokines. Interestingly, the authors did not find the described changes in men’s heart tissues [96].

Although aging is associated with a decrease in AMPK and sirtuins, cancer is more likely to exhibit an increase in activity, which may have both pro- and anticancer effects. In the absence of cancer, before a tumor develops, AMPK activity has an anti-tumor effect. However, if cancer has already developed, AMPK promotes further tumor growth as it enhances cancer cell survival, metabolism and proliferation [97,98].

Similarly, higher levels of sirtuins were observed in various cancer cell lines and are associated with disease severity, poor prognosis, increased angiogenesis and resistance to therapy [99,100,101].

Thus, it is evident that aging and cancer are associated with changes in nutrient-sensing pathways and that altering them may have a therapeutic effect. Increasing the activity of nutrient-sensing pathways may slow aging, whereas reducing their activity may limit tumor development.

## 5. Intercellular Communication

Intercellular communication includes all agents and molecules (soluble and membranes) that are produced by cells. As we have already described, aging cells undergo significant changes that lead to changes in the expression of surface and soluble molecules. Typically, we see this in the SASP phenotype, which is associated with senescence or inflammaging. Therefore, the dynamics of intercellular communication changes during aging [102,103,104].

IL-6 is often evaluated in the context of aging and intercellular communication. IL-6 is involved in various processes, including inflammation, autoimmune reactions, metabolism and cancer, and its levels increase during aging and in the presence of cancer [105,106].

In this respect, it is very difficult to focus on certain molecules. For example, IL-6 detection is widely used. However, we will look at extracellular vesicles. These are secretory double membrane vesicles that contain the cargo produced by cells, reflecting their transcriptomes, proteomes and metabolomes, which are different in aged and cancer cells compared with young, healthy cells.

They are found in all body fluids and can interact with cellular receptors, as well as endocytose, and affect intracellular processes. EVs not only transmit signals to the immediate environment but, together with the flow of fluids such as blood, they travel from the parent cell to the whole body. EVs can modulate all the mentioned hallmarks of aging, including genetic hallmarks such as telomere length, genomic stability and epigenetics [107].

Studies show that aging is associated with a decrease in EV production, although senescent cells and cells exposed to pro-inflammatory stimuli (age affects immune function and there is a shift towards inflammatory responses) produce higher numbers of EVs. However, there appears to be an increase in EV endocytosis, resulting in their levels not being elevated [108].

Eitan et al. examined the levels of EVs in participants divided into groups according to age (30–35; 40–55; 55–64 years) and found that EV levels decreased with age, whereas internalization of EVs by B cells increased. EVs endocytosed by monocytes increased MHC II expression, especially EVs from older donors [109]. There are specific changes in the cargo composition in aged EVs. For example, aged-cell EVs express a lower level of galectin 3 and mitochondria and their components [110,111]. Interestingly, expression of CD63 in EVs differs in the cerebrospinal fluid (CSF) and plasma with age, as shown by de Andrade et al. in a mouse model [112]. Whereas the expression increases in the CSF, it decreases in plasma. In plasma, levels of IL-1β increased with age. Geotzl et al. showed that platelets from older subjects express a higher level of alarmin HMBG1 (high mobility group box 1) [113].

Non-coding mRNAs have good potential as biomarkers of aging in EVs [114,115]. D’Anca et al. defined sets of miRNAs related to physiological aging and sets related to age-related degenerative diseases [116]. One of the age-related mRNAs is, according to Machida et al., miR-24-3p, which is positively correlated with age [117]. As we know, during aging, the number of senescent cells that produce specific EVs increases. Mensà et al. revealed that these EVs have elevated levels of miRNA-21-9 and miR-271, which target SIRT1 and DNA (cytosine-5)-methyltransferase 1 and induce senescence in other cells, particularly in the endothelium [118].

We could go on and on with the list of miRNAs and other components of EVs; it is a very broad topic.

In cancer, the intensity of production and the cargo of EVs differs from healthy cells. The levels of EVs increased in both in vivo models and clinical trials [119]. Matsumoto measured EV levels in patients with esophageal squamous cell carcinoma. The levels were higher in cancer patients than in noncancer patients but there was no correlation between EV levels and tumor progression [120]. EVs can serve as a biomarker of tumor progression, especially EVs expressing specific molecules [121]. Non-small cell lung carcinoma is associated with increased production of EVs expressing miRNA-126, which are capable of inducing angiogenesis and malignant transformation in human bronchial cells [122].

In patients with breast cancer, elevation of the levels of EVs expressing miRNA-182 was documented, whereas EVs from patients with laryngeal squamous cell carcinoma expressed higher levels of miRNA-941, which in cell culture promoted cell proliferation and invasion [123,124]. Liu et al. identified exosomal miRNA-139-3p as a biomarker of colorectal cancer. Its expression was significantly decreased in patients compared with healthy controls, and there were even lower levels of exosomal miRNA-139-3p in patients with metastatic cancer [125].

In both aging and cancer, changes in intercellular communication led to the progression of aging and age-related diseases in aging, whereas in cancers it promoted their progression. By targeting this hallmark, as well as others mentioned previously, it is possible to intervene in aging and promote cancer treatment.

## 6. Immune System Dysfunction

Aging and tumorigenesis are associated with changes in immune system reactivity. Changes in the immune system can be both a cause and a consequence of ongoing processes and drive and accelerate pathological processes. During aging, even healthy aging, there are typical changes in the immune system that are termed collectively as inflammaging and immunosenescence. Senescence is a pro-inflammatory factor, as are changes in the immune system that occur in both innate and adaptive immunity and lead to low-grade inflammation, altered resolution of inflammation, increased susceptibility to autoimmune reaction, suppressed anti-infective and antitumor immunity [126].

As the years progress, there are changes in the number of immune cells. Aging is accompanied by an alteration in bone marrow function and a decrease in hematopoiesis. Interestingly, it is mainly the production of lymphoid cells (T, B and NK cells) that is affected by aging, whereas the production of myeloid lineages (monocytes, neutrophils) remains unchanged or even increases [127].

### 6.1. Aging and Adaptive Immunity

In adaptive immunity, the changes affect both T and B lymphocytes. The production and proper development (training) of T and B cells are significantly altered. Training of T cells depends on the thymus, which undergoes progressive atrophy, whereas training of B cells occurs in the bone marrow. By training, we mean the ability of T and B cells to recognize foreign antigens and tolerate self-antigens. Disrupted elimination of autoreactive T and B cells leads to autoimmune diseases [128].

Typical changes in the population include a decrease in the number of naive T cells with a concomitant increase in the number of memory phenotypes, which is associated with a decrease in the repertoire of TCRs (diversity of TCRs). The ability to differentiate into different subsets and activation is also limited. The main subsets that increase in number are Th1, Th17 and Treg cells, whereas the number of CD8^+^ cells rapidly decreases [129,130,131].

Unconventional T cells, γδ T cells, iNKT cells and MAIT cells, decline rapidly with age and their proliferative capacity and susceptibility to apoptosis is enhanced. However, the numbers and activity of some subsets of these cells may be increased, e.g., only in some organs. Changes in the number of these cells are also sex dependent [132].

In terms of B lymphocytes, the most important changes during aging are a decrease in B lymphocyte production, a decrease in their response to stimuli and a decrease in antibody production [133,134].

### 6.2. Aging of the Innate Immunity

In innate immunity, changes in the number of cells are not so significant; however, their functions are altered. Changes affect all representatives of innate cellular immunity. Cytotoxic NK cells show a decrease in cytotoxicity and therefore in the ability to remove damaged, infected or tumor cells [135].

Monocytes and macrophages differentiate into proinflammatory subsets, whereas phagocytosis, the degradation of engulfed material, antigen presentation and the ability to stimulate T lymphocytes decrease. We cannot omit the fact that macrophages have a lower capacity to terminate and resolve inflammation [136,137].

Hearps et al. showed that older monocytes have upregulated expression of activation markers such as CXCL10, neopterin and sCD163. They also exhibit impaired phagocytosis, shortened telomeres and increased concentration of intracellular TNFα after stimulation with TLR4 agonist [138].

Aging also affects dendritic cells, which present of antigens, prime T cells and produce large amounts of cytokines. In the elderly, the number of conventional dendritic cells is lower than in younger people, and the levels of plasmacytoid dendritic cells remain stable. However, the production of IFNγ, TNFα, IL-6, IL-12 and IL-10 and phagocytosis is altered. IL-12 is crucial for the development and function of NK and T cells [139].

Other important cells in innate immunity are neutrophils, which phagocytose and kill pathogens, recruit and activate dendritic cells, monocytes and lymphocytes and migrate to the site of damage. Neutrophil counts do not fluctuate significantly in the elderly. However, there is a decrease in chemotaxis, phagocytosis and the destruction of engulfed pathogens as oxygen radical production is reduced. Neutrophils are also more susceptible to apoptosis [140,141].

Mast cells, eosinophils and basophils are involved not only in hypersensitive reactions but also in maintaining homeostasis in the mucosal layer and protection against infection. In these cell types, activation and degranulation decrease with aging; however, numbers can increase in distinct locations [142,143]. The number of mastocytes increases in tissues, whereas the number of basophils in mice is elevated in the bone marrow and spleen and the cells exhibit a changed phenotype [144]. Mathur et al. described changes in eosinophils in participants with asthma. They found that degranulation in response to IL-5 stimulation and superoxide anion production by eosinophils decreased in older patients. Thus, they confirmed that eosinophils undergo age-dependent changes [145].

We cannot fail to mention, at least briefly, innate lymphoid cells. Studies with human cells are lacking but through mouse studies we know that aging also affects innate lymphoid cells, especially ILC2 cells. They are located in the tissue and epithelium and support the maintenance of mucosal barrier function. ILC2 cell production increases but the number of ILC2 cells in peripheral tissue (airways) decreases. It is documented that their function also declines. Transfer of rejuvenated ILC2 cells to the aged brain leads to an increase in cognitive function [146,147].

### 6.3. Aging and Humoral Immunological Parameters

During aging, not only are the cells of the immune system affected but also the humoral components, that is, the complement. The complement system consists of nine proteins that activate each other in a cascade and are involved in the antimicrobial protection and activation of other immune responses. Since many changes and damage occur during aging, the complement system can be overactivated. According to studies, C3 and C4 levels correlate with age. In the case of longevity, this correlation is negative. The C3/C4 ratio is important; higher levels of C3 are thought to “shorten” life. Complement is involved in many disorders typical for older age, such as macular degeneration, Alzheimer’s disease, cardiovascular disease and osteoarthritis [148].

Inflammation, however mild, remains ongoing in the body and is a source of oxidative stress and tissue damage. This is associated with higher levels of TNFα, IL-1β, IL-6 and CRP, etc. Subsequently, these changes accelerate the process of aging, the development of frailty syndrome and (even in younger people) neurodegenerative, cardiovascular and autoimmune diseases and cancer [149,150,151].

### 6.4. Cancer and Immune System

The main players in antitumor immunity are cytotoxic cells: NK and CD8^+^ T cells. These cells directly kill tumor cells and release cytokines that activate other immune cells. Antigen-presenting cells, mainly dendritic cells, are also involved in the immune response to cancers and induce adaptive immune reactions, including the production of specific antibodies.

The aged immune system and increased inflammatory activity cannot control tumor cells, which, in addition, influence the activity of the immune system to their advantage and evade immune system surveillance. The tumor microenvironment can inhibit immune cell activity and induce the differentiation of immune cells into inhibitory and tolerogenic phenotypes (tolerogenic DC, T and B regulatory cells, M2 macrophages) and trigger immune cell apoptosis and senescence [152,153].

The presence of cancer further increases inflammation [154]. Inflammation and immunosenescence are therefore risk factors for cancer. On the other hand, the presence of cancer and anticancer treatments in children and young people can trigger low-grade inflammation—inflammaging—that can induce premature aging and damage to vital organs [155,156].

### 6.5. Clinical Immune Markers of Aging and Cancer

Martínez de Toda et al. measured the parameters of immune function in three groups of volunteers: adult (30 to 49 years), mature (50 to 64 years), old (65 to 79 years), and long-lived (90 to 103). They isolated lymphocytes, neutrophils, NK cells and macrophages and evaluated their functions. Functions, such as activity, proliferation, phagocytosis and chemotaxis declined with age; however, in long-lived groups, the parameters were more similar to those of adults. The function remained preserved. Therefore, maintaining immune function is a factor that can prolong life [157].

The presence of low-grade inflammation was documented by Puzianowska-Kuźnicka et al., who analyzed blood samples from 4979 individuals ≥65 years old. IL-6 levels increased with age and healthy aging was associated with lower levels of CRP and IL-6 compared with aging with chronic diseases. CRP and IL-6 levels are good predictors of mortality risk and chronic disease in the elderly [158]. Milan-Mattos et al. also measured IL-6 and hsCRP and added TNFα. They revealed a positive correlation between age and hsCRP and IL-6. The increase was more pronounced in the group with individuals aged 51 to 60 years. Gender differences were found, with older women having higher levels of hsCRP and IL-6 compared with similarly aged men [159].

Evidence that IL-6 plays a crucial role in inflammaging and could be a potential therapeutic target was provided by Squarzoni et al. They showed that neutralization of IL-6 can ameliorate symptoms in progeroid mice. The application of tocilizumab stabilized nuclear envelope chromatin and the limited hyperactivated DNA damage response in vitro and in vivo led to a reduction in aortic lesions and adipose tissue dystrophy, prevented motor disability and improved life quality [160].

The increase in IL-6 and CRP is typical for inflammaging, and both proteins are also elevated in cancer and their levels are associated with tumor progression and prognosis. This was confirmed, for example, in patients with colorectal cancer and melanoma [161,162,163].

## 7. Stem Cell Exhaustion

Stem cells are a unique cell type that are capable of self-renewal, proliferation and differentiation into multiple cell types due to their potency (monopotent, multipotent). Stem cells are found in all tissues and are involved in regeneration processes and, in the case of hematopoietic stem cells, hematopoiesis. However, during life, stem cells are gradually depleted and exhausted and lose their regenerative potential and clonogenity [164]. Their damage is mainly due to mitochondrial disruption, shortening of the telomere, DNA damage, induction of senescence, disruption of proteostasis and the influence of the microenvironment in which the cells are located [165,166].

### 7.1. Stem Cells and Aging

Stem cell aging is accepted as confirmed, as we also see in the case of donor selection for transplantation. For hematopoietic stem cells, donor ages range from 18 to 40 years. Since mesenchymal stem cell therapy is not part of standard treatment but is part of experimental or clinical trials, the age of the donors is not determined [167].

Aged hematopoietic cells preferentially differentiate into myeloid cells, which are related to the increased incidence of myeloid leukemia in the elderly compared with younger people, in whom lymphoid types of hematologic malignancies are more common [168].

Studies show that, in vitro, aged mesenchymal cells preferentially differentiate into certain cell lines, such as adipocytes [169].

Zaim et al. conducted a study with individuals ranging in age from 0 to over 60 years of age. They found that the potential for adipogenic, osteogenic and neurogenic differentiation declines with age, whereas chondrogenic differentiation is preserved [170].

Stem cells are not only exhausted but can also transition into a state of senescence. Senescent stem cells undergo morphological and, more importantly, functional changes. The most typical is a significant decline in proliferation and migration.

Although young stem cells have more immunomodulatory and anti-inflammatory potential, older senescent cells have an SASP and produce more pro-inflammatory agents. An SASP can drive the premature senescence of neighboring cells [171,172].

It is obvious that, in the case of aging, it is important to maintain stem cells in a condition to prevent the decline in the number of cells and their functions.

### 7.2. Stem Cells and Cancer

Cancer stem cells are a small heterogeneous tumor cell population that can initiate tumor development. Cancer stem cells can arise either from stem cells or by the differentiation (dedifferentiation) of cancer cells that acquire stemness. They are immortal and have higher proliferative potential, higher resistance to treatment and lower immunogenicity. They are a cause of relapse after treatment [173]. Anticancer treatment can induce senescence in healthy and cancer cells; however, cancer stem cells can escape senescence. They can undergo other additional mutations that can deactivate tumor suppressors, e.g., p53, p16, p19ink4d, and overexpress CDC2/CDK1. Senescent cells can also experience this process and gain stemness [172,174]. For anticancer treatment to be successful, it is necessary to target cancer stem cells and block senescence-associated stemness [175].

It is obvious that, in the case of aging, it is important to maintain stem cells in a condition to prevent the decline in the number of cells and their functions, whereas in the case of cancer stem cells, it is necessary to eliminate these cells.

## 8. Mitochondrial Dysfunctions

**Figure 6:**
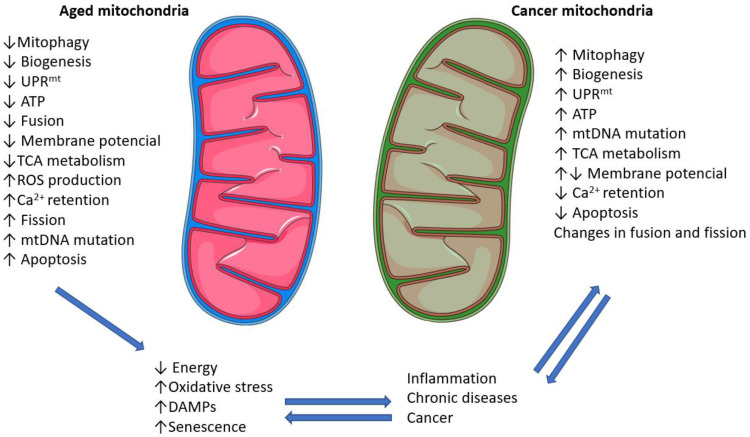
Difference between aged and cancer mitochondria. Legend: Comparison of the differences between mitochondria from normal cells and mitochondria from aged and cancer cells. Changes in mitochondrial function lead to changes that can be bidirectional (arrows). Abbreviations: UPR^mt^, mitochondrial unfolded protein response; ATP, adenosine triphosphate; TCA, tricarboxylic acid; ROS, reactive oxygen species; DAMP, danger associated molecular pattern.

Mitochondria are intracellular organelles that produce energy. They are involved in intracellular signaling; they use reactive oxygen species (ROS), regulate cellular metabolism and induce apoptosis. Mitochondrial DNA (mtDNA) is a maternally inherited autonomous genome of mitochondria. Mitochondria are found in large numbers in the cell. mtDNA encodes 13 OXPHOS proteins, which are part of the enzyme complex in the respiratory chain, 22 tRNAs and 2 rRNAs. During aging, the number, morphology and functions of mitochondria change (Figure 6).

### 8.1. Morphological Changes in Mitochondria in Aging and Cancer

The mitochondria in aged cells tend to be more fragmented, circular, shorter and smaller. Hyperfused mitochondria occur more frequently and there are defects in mitochondrial division and mitochondrial biogenesis. Although mitochondrial biogenesis is reduced, the total number of mitochondria in the cell increases. This is due to reduced mitophagy, by which damaged mitochondria are removed [176,177].

The accumulation of altered mitochondria is due not only to a higher incidence but also to a reduction in their removal by mitophagy (an autophagic process that removes mitochondria), which maintains the homeostasis of these organelles in the cell [178]. The mitochondria in a tumor cell must adapt to a change in environment, with less oxygen and more energy demand. There are changes in morphology and function. Changes include reduction of elongation, the filamentous phenotype changing into aggregated and mitochondria being localized mainly perinuclearly; therefore, the mitochondrial network morphology is significantly disrupted and the membrane potential decreased [179,180].

### 8.2. Changes in Mitochondrial DNA (mtDNA) in Aging and Cancer

Morphological changes are accompanied by a higher incidence of mutations in mtDNA. As the years pass, mtDNA heteroplasmy occurs, where both normal and mutant mtDNAs are present in the cell and the proportion of mutant mtDNA gradually increases [181]. The presence of mutant mtDNA is associated with a decrease in mitochondrial DNA polymerase activity or dysfunction [182,183]. In addition to mtDNA mutations, the decline in mtDNA levels plays significant roles in chronic diseases such as diabetes, overweight and obesity [184].

As with aging, mutations in mtDNA occur in cancer cells. Deletions and single nucleotide polymorphisms of mtDNA are widespread in various tumor types and are often associated with OXPHOS deficits. Changes in mtDNA are also associated with tumor invasivity and metastasis [185].

### 8.3. Changes in Respiratory Chains in Aging and Cancer

Functional changes in mitochondria during aging include decreased respiratory chain efficiency, increased ROS production and decreased ATP production. This is due to the disruption of the membrane potential and the reduction of OXPHOS activity and thus the activity of individual protein complexes in the respiratory chain. This defect is often caused by mutations in mtDNA. Bowman et al. confirmed electron transport chain dysfunction and increased ROS production, but not all cells are affected. They analyzed human fibroblasts and keratinocytes from donors aged 6 to 72 years. Complex II was significantly lower in aged fibroblasts but not in keratinocytes [186].

Boffoli et al. proved that this decline also affected skeletal muscle cells. They measured respiratory chain activity in the skeletal muscles of participants aged 17 to 91 years and found that the activity of complexes I, II and IV decreased with age [187]. The decline in respiratory chain was also documented in the crypts in the colon and intestinal mucosa [188,189].

In cancer cells, the production of ROS is limited, since low and moderate levels drive cell proliferation. The products of oncogenes (Ras, myc) regulate respiratory chain activity. Wall et al. showed that the transcription factor Singleminded-2, which facilitates the function of the respiratory chain, is deleted in breast cancer cells [190,191].

### 8.4. Aging and Mitochondrial NAD^+^

Mitochondrial dysfunction can be detected using many markers, as we showed in the previous text: mtDNA, OXPHOS proteins, ROS production and other markers including GDF-15, mitomiR (miRNA localized within mitochondria) and NAD^+^.

In mitochondria, NAD^+^ plays a crucial role in the tricarboxylic acid cycle, oxidative phosphorylation and ATP production. NAD^+^ is synthesized in the cell cytosol and transported to the mitochondria. In mitochondria, NAD^+^ accepts free electrons that are formed during its metabolic processes. Thus, proper mitochondrial function depends upon a sufficient quantity of NAD^+^ [192,193].

NAD^+^ levels have been evaluated in relation to aging in many studies. The key finding is that there may be a decline during aging. In the context of aging, it should be mentioned that NAD^+^ regulates sirtuin activity, which is discussed in the text on nutrient sensing.

NAD^+^ levels can be detected both in tissues and in plasma. It has been proven that the levels of NAD^+^ decline with age in plasma, skin or the brain [194,195,196]. Karas et al. also confirmed age-dependent reduction in NAD^+^ levels and the negative correlation of this with AGEs [197].

In cancer cells, the levels of NAD^+^ are higher than in healthy cells [198]. Increased levels of NAD^+^ are associated with enhanced DNA repair capacity and the resistance to chemotherapy [199].

### 8.5. GDF-15, a Marker of Mitochondrial Stress, in Aging and Cancer

GDF-15 (growth/differentiation factor 15) can serve as a marker of mitochondrial dysfunction. Its levels are zero or very low during adulthood and increase during aging or in the presence of chronic diseases, since its expression is induced by cellular stress, inflammation and oxidative stress. Elevated levels of GDF-15 in children and young adults are associated with mitochondrial diseases such as mitochondrial myopathies [200]. Elevated levels in adults are associated with myopathies and muscle weakness [201].

Liu et al. evaluated GDF-15 levels in participants of different ages (23- to 83-year-old males). The results showed that GDF-15 levels were higher in older participants and positively correlated with age. The authors suggested that GDF-15 is a potential biomarker of aging [202].

Welsh et al. measured serum GDF-15 concentrations in 19,462 participants and set reference values for specific age groups according to the measured values. The authors also confirmed the association of GDF-15 levels with diabetes and cardiovascular disease (stroke, heart failure) and their markers such as cardiac troponins I and T and N-terminal pro B-type natriuretic peptide [203].

GDF-15 has both a pro-tumorigenic and anti-tumorigenic effects. In certain types of tumors, the values reach 200 times the expected values (melanoma, breast, colorectal, pancreatic, cervical or prostate cancer) [204,205]. Vocka et al. suggested that GDF-15 can serve as a prognostic marker in patients with metastatic colorectal cancer. GDF-15 levels were significantly higher in patients than healthy controls and were correlated with the severity of the disease [206]. Suzuki et al. tested GDF-15 levels in patients with advanced pancreatic cancer. The cut-off level was determined to be 3356.6 pg/mL. In the high GDF-15 group, there were patients with more severe conditions, inflammation, loss of appetite and cachexia [207].

### 8.6. Noncoding Mitochondrial RNA in Aging and Cancer

Other interesting markers of mitochondrial dysfunction are noncoding RNAs, mitomiRNAs. They are mostly cytoplasmic RNAs that enter the mitochondria and orchestrate mitochondrial functions. Therefore, there is a difference between the composition of cytosolic and mitochondrial miRNAs. Although several mitomiRNAs originate in the nucleus, there are some that are coded from mtDNA. The composition and expression of mitomRNAs change during aging and disease. Several of them are involved in aging, inflammaging and cancer [208].

Numerous mitomiRNAs play a role in aging and age-related diseases [209,210]. However, there is not the space to list individual miRNAs but rather to inform the reader about their existence and their connection to aging. Unsurprisingly, changes in mitomiRNAs are also found in cancer cells. Several mitomiRNAs are responsible for cancer development and metastasis, e.g., miRNA-195 is associated with breast cancer, miR-210-5p with colon cancer, miRNA-125a is expressed in cisplatin-resistant laryngeal cell carcinoma and miRNAs 17 and 20 in chemoresistant leukemia [211]. These mitomiRNAs can change mitochondrial genes and reprogram mitochondrial metabolism, respiratory chain activity and the resistance to apoptosis [211,212].

There is a close link between mitochondria, aging and carcinogenesis. The individual steps potentiate each other. The question is, as with other hallmarks of aging, whether damage or alteration of mitochondrial function is a cause or a consequence.

## 9. Dysbiosis

**Figure 7:**
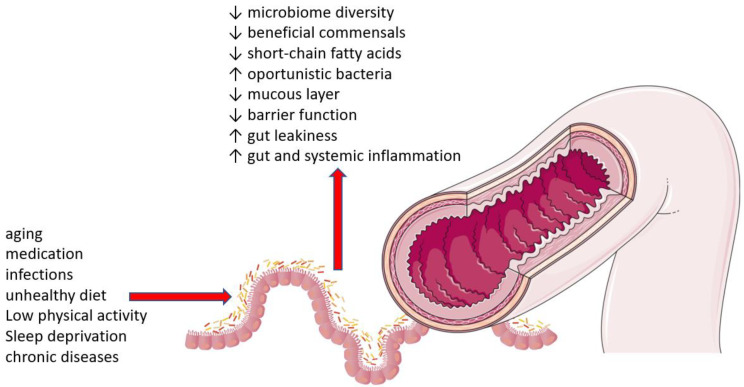
Gut microbiota environment. Legend: the composition of the microbiota is influenced by various factors (horizontal arrow), which leads to changes in the microbiota (vertical arrow); SCFA, short-chain fatty acid.

The microbiota is the community of microorganisms (bacteria, viruses, fungi and parasites) that affect human health. Microorganisms inhabit all parts of our body, and each area has a different composition of microbiota. The most studied is the gut microbiota. The composition of the microbiota can vary during life. It is influenced by lifestyle, for example, diet, medications (especially antibiotics), physical activity, sleep and circadian rhythms, the use of cosmetics and the presence of chronic diseases. Aging also plays an important role (Figure 7) [213,214]. Aging is associated with a microbiome disturbance, dysbiosis. The main feature of this process is the shift in microbial populations and the loss of their diversity. The main aging-related changes include the loss of dominant commensal taxa such as *Faecalibacterium*, *Prevotella*, *Eubacterium*, *Lachnospira* and *Bifidobacterium* and the increase in commensal taxa such as *Akkermansia*, *Butyricimonas*, *Odoribacter* and *Oscillospira* and pathobionts such as *Streptococcus, Bilophila*, *Eggarthella*, *Escherichia*, *Fusobacterium* and *Clostridum*. Interestingly, centenarians have typical patterns of microbiome composition. Certain bacterial species seem to promote longevity [215]. Importantly, young adults and centenarians have higher microbial diversity than the elderly, especially if they have an associated chronic disease [216,217].

Refs. [216,217]. Wilmanski et al. conducted a study involving 9000 participants and showed that aging is associated with distinct changes in the microbiota that differ between healthy aging and aging associated with the presence of disease. Healthy aging leads to the depletion of core genera, especially *Bacteroides*. The absence of a natural decline in *Bacteroides* during aging or a significant decrease in diversity reduced survival over the 4-year period the study lasted [218].

Various studies have evaluated the effect of the microbiota and its changes on the development and progression of different types of cancer. The results show that the microbiota plays an important role in oncogenesis. Changes in its composition can induce inflammation, oxidative stress, DNA damage to cells, etc. Some pathological bacteria found in the disrupted microbiota are directly linked to cancer, e.g., *Helicobacter pylori* is linked to gastric adenocarcinoma. *Chlamydia trachomatis*, *Chlamydia pneumonia*, *Escherichia coli*, *Bacteroides fragilis* and *Salmonella enterica* can be involved in cervical, lung, colorectal and gallbladder cancers [219,220].

Modulation of the composition of the microbiota may act to prevent cancer. Zhang et al. revealed that the presence of tissue-resident *Lachnospiraceae* family bacteria protects against colorectal carcinoma. Bacteria maintain and facilitate the immune surveillance functions of CD8+ T cells [221].

Therefore, the composition of the microbiota, its diversity and the number of individual bacterial species play an important role in both the aging process and the pathogenesis of cancer. Influencing its composition has therapeutic potential.

## 10. Conclusions

Aging is an inevitable process. It is a gradual decline that causes changes in the many processes taking place in the body. These changes occur at different rates according to the genetic predisposition of the individual but are largely due to the individual’s lifestyle and the influence of the external environment. Changes associated with aging predispose individuals to the development of cancer.

In this review article, we have described the most important non-genomic hallmarks of aging and the research studies aimed at elucidating these hallmarks, how they function and how they can be influenced and be beneficial in clinical practice, in procedures to slow down aging and in the treatment of age-related diseases such as cancer.

## Data Availability

Not applicable.
